# Supplementary material for: Pre-analytical drivers of bias in bead-enriched plasma proteomics
Source: EMBO Mol Med. 2025 Sep 12;17(11):3174–96. doi: 10.1038/s44321-025-00309-0 (PMC12603263; doi:10.1038/s44321-025-00309-0)
Supplement: Supplementary file 1 — Appendix [file 44321_2025_309_MOESM1_ESM.pdf]

Appendix Figures

Figure	Title	Page
Appendix Figure S1	Analysis of bead-detected proteins and quantitative precision	2
Appendix Figure S2	Validation and characterization of cell-specific quality markers	5
Appendix Figure S3	Enrichment scores of cell-specific markers across contamination series	6
Appendix Figure S4	Workflow-specific contamination marker analysis	7
Appendix Figure S5	Overlap of identified yeast proteins between workflows at different dilution ratios	10
Appendix Figure S6	Dilution series analysis of yeast proteins across abundance ranges	12
Appendix Figure S7	Analysis of protein enrichment across yeast protein abundance categories	13
Appendix Figure S8	Platelet marker analysis across bead-buffer combinations in platelet-contaminated samples	14
Appendix Figure S9	Enrichment/depletion analysis before and after 30-minute centrifugation	15
Appendix Figure S10	Contamination markers across centrifugation conditions	16

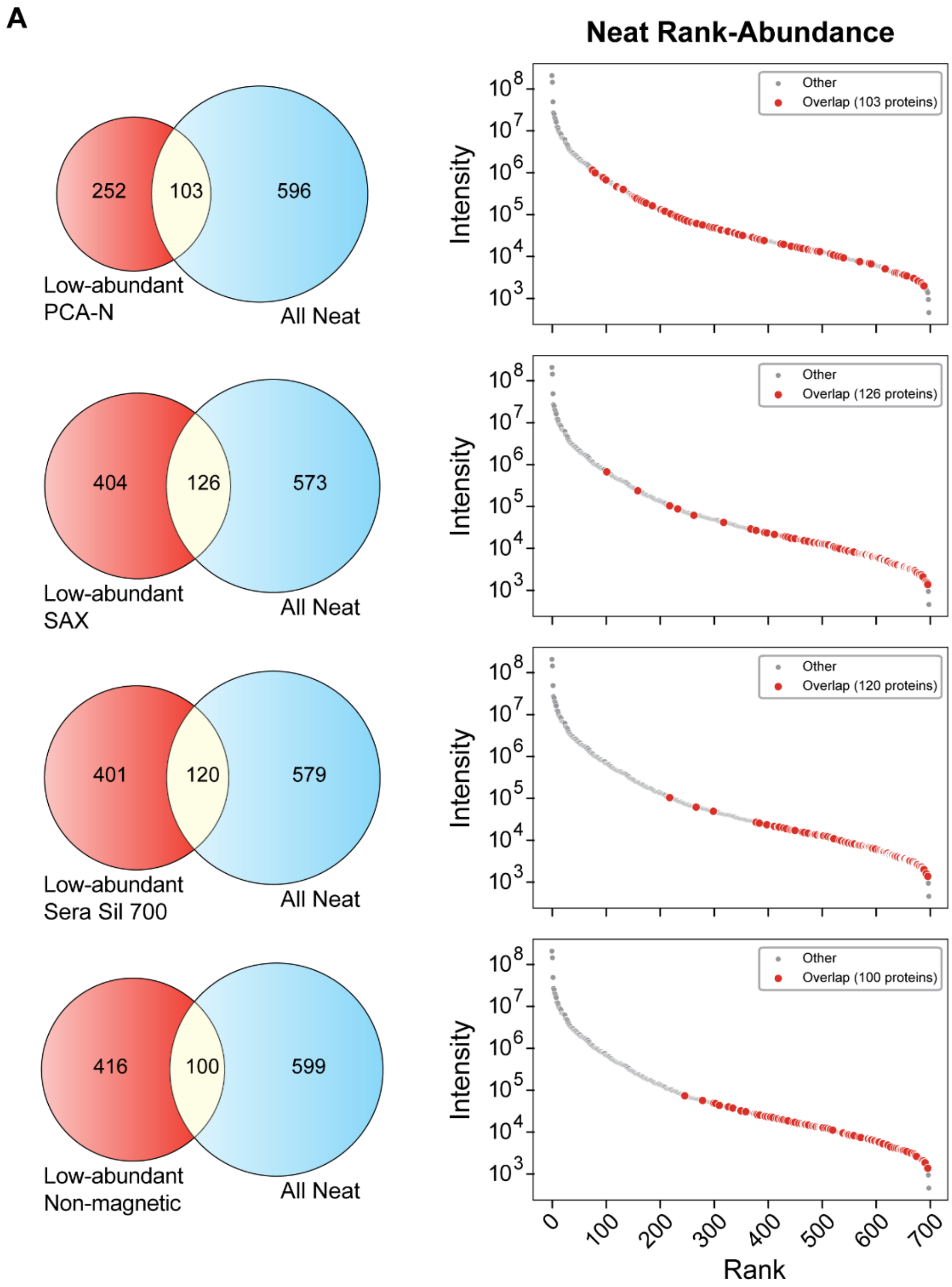
A
Neat Rank-Abundance
252
103
596
Low-abundant
PCA-N
All Neat
404
126
573
Low-abundant
SAX
All Neat
401
120
579
Low-abundant
Sera Sil 700
All Neat
416
100
599
Low-abundant
Non-magnetic
All Neat
Intensity
Other
Overlap (103 proteins)
Overlap (126 proteins)
Overlap (120 proteins)
Overlap (100 proteins)
Rank

B

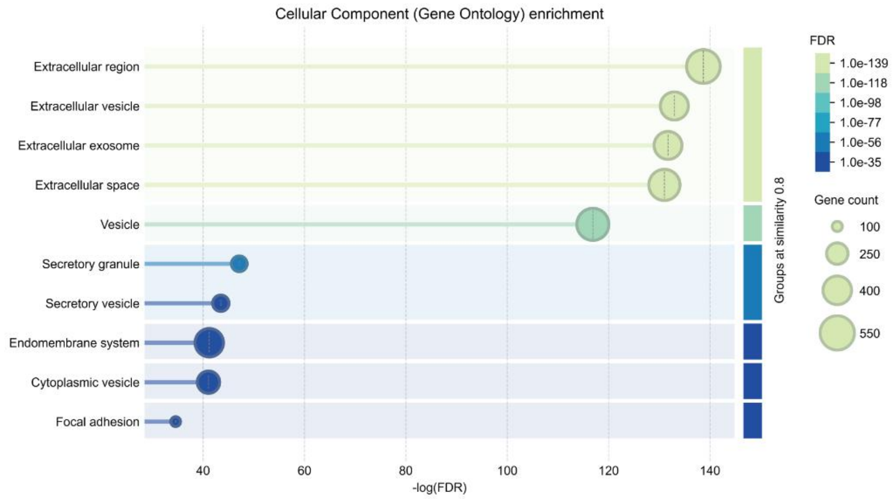
Cellular Component (Gene Ontology) enrichment
Extracellular region
Extracellular vesicle
Extracellular exosome
Extracellular space
Vesicle
Secretory granule
Secretory vesicle
Endomembrane system
Cytoplasmic vesicle
Focal adhesion
40
60
80
100
120
140
-log(FDR)
Groups at similarity 0.8
FDR
1.0e-139
1.0e-118
1.0e-98
1.0e-77
1.0e-56
1.0e-35
Gene count
100
250
400
550

C

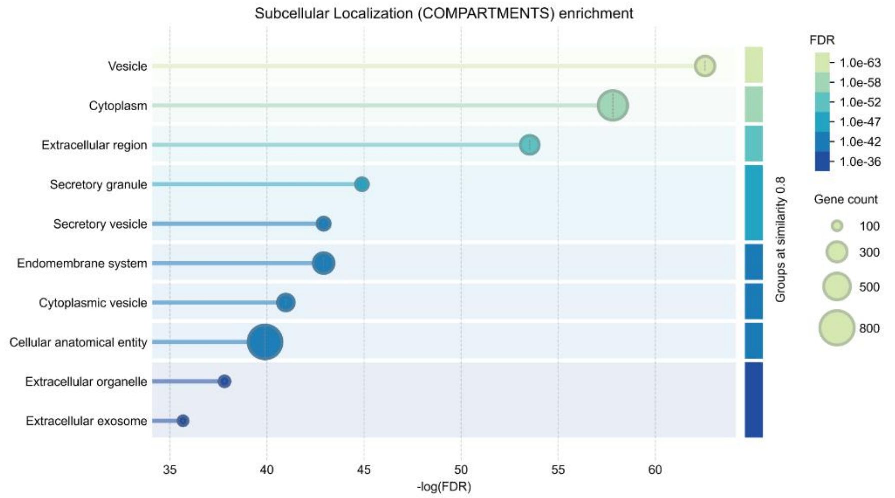
Subcellular Localization (COMPARTMENTS) enrichment
Vesicle
Cytoplasm
Extracellular region
Secretory granule
Secretory vesicle
Endomembrane system
Cytoplasmic vesicle
Cellular anatomical entity
Extracellular organelle
Extracellular exosome
35
40
45
50
55
60
-log(FDR)
Groups at similarity 0.8
FDR
1.0e-63
1.0e-58
1.0e-52
1.0e-47
1.0e-42
1.0e-36
Gene count
100
300
500
800

D

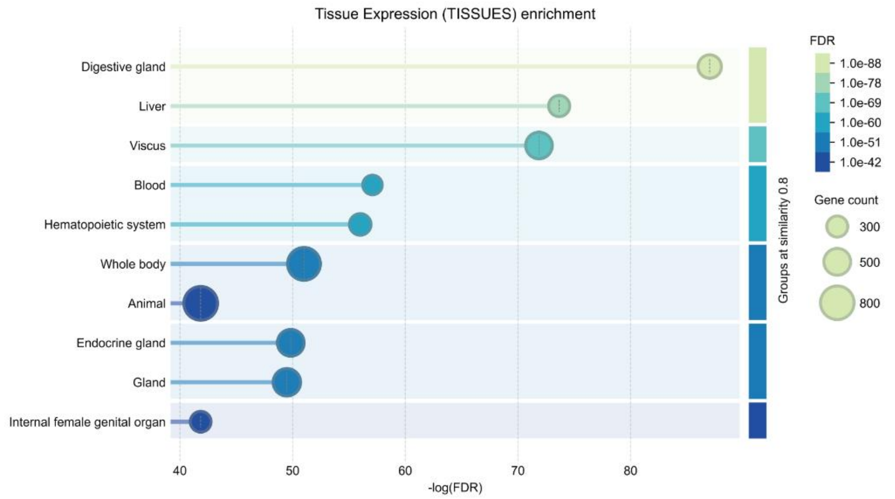
Tissue Expression (TISSUES) enrichment
Digestive gland
Liver
Viscus
Blood
Hematopoietic system
Whole body
Animal
Endocrine gland
Gland
Internal female genital organ
40
50
60
70
80
-log(FDR)
Groups at similarity 0.8
FDR
1.0e-88
1.0e-78
1.0e-69
1.0e-60
1.0e-51
1.0e-42
Gene count
300
500
800

E

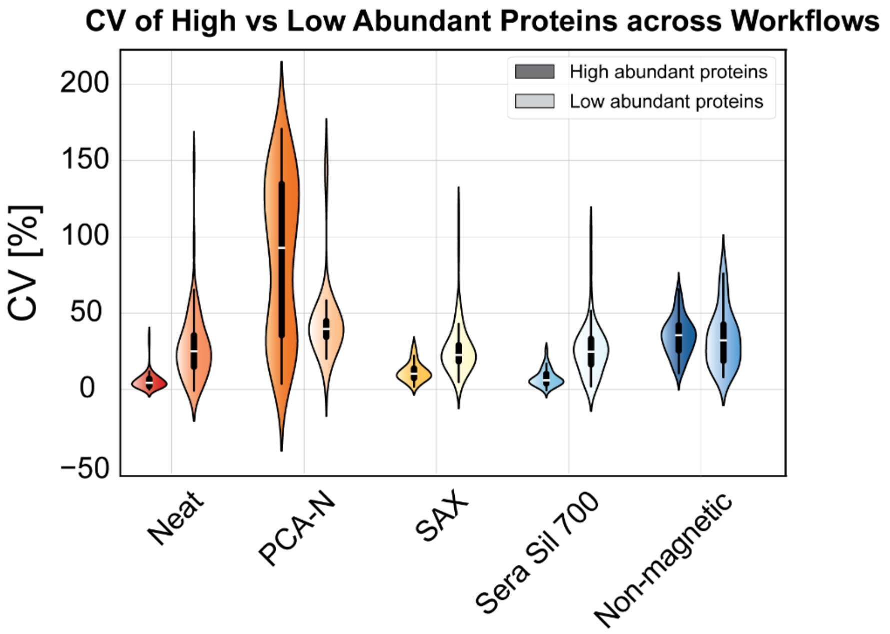

Appendix Figure S1 - ***Analysis of bead-detected proteins and quantitative precision***

(A) Overlap analysis of low-abundance proteins between workflows. Venn diagrams and rank abundance plots showing overlap between low-abundance proteins detected by each workflow and all proteins identified in neat plasma. Right: Neat plasma rank abundance plots with overlapping proteins highlighted in red.

(B-D) Functional enrichment analysis of proteins uniquely detected by bead-based methods. Enrichment analysis of approximately 850 proteins uniquely detected by bead-based workflows compared to neat plasma. (B) Cellular component analysis. (C) Subcellular localization analysis. (D) Tissue expression analysis. Dot size represents gene count, color intensity indicates statistical significance (FDR).

(E) Quantitative precision comparison across workflows. CV analysis comparing high-abundance proteins and low-abundance proteins as defined by neat plasma ranking. Violin plots show CV distributions across all five workflows.

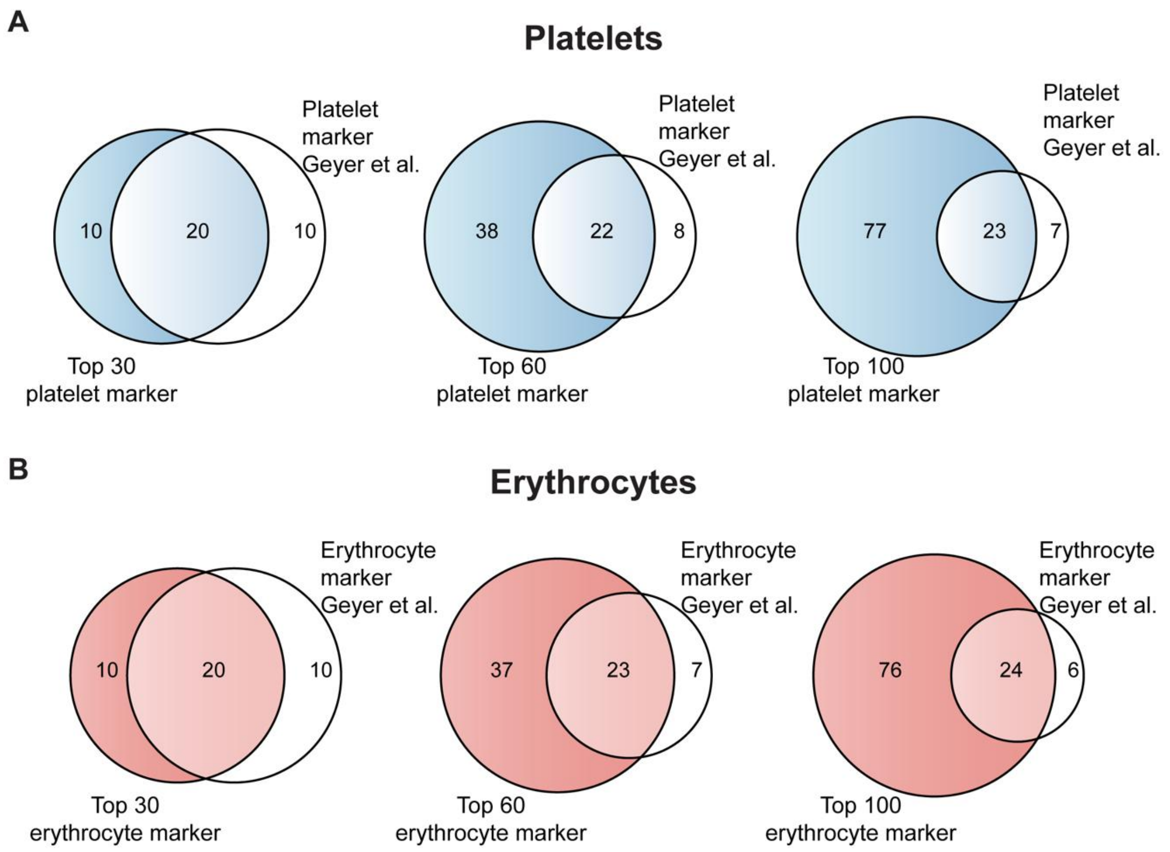

*Appendix Figure S2 - **Validation and characterization of cell-specific quality markers**.*

(A-B) Comparison of identified quality markers with previously published markers from Geyer et al. Venn diagrams show overlap between

(A) platelet markers and (B) erythrocyte markers at three different stringency levels (top 30, top 60, and top 100 proteins). Despite differences in analytical platforms and experimental setup, substantial overlap is observed, with 67% identity (20 proteins) for both cell types in the top 30 panel, increasing to 22-23 proteins in the top 60 and 23-24 proteins in the top 100 markers.

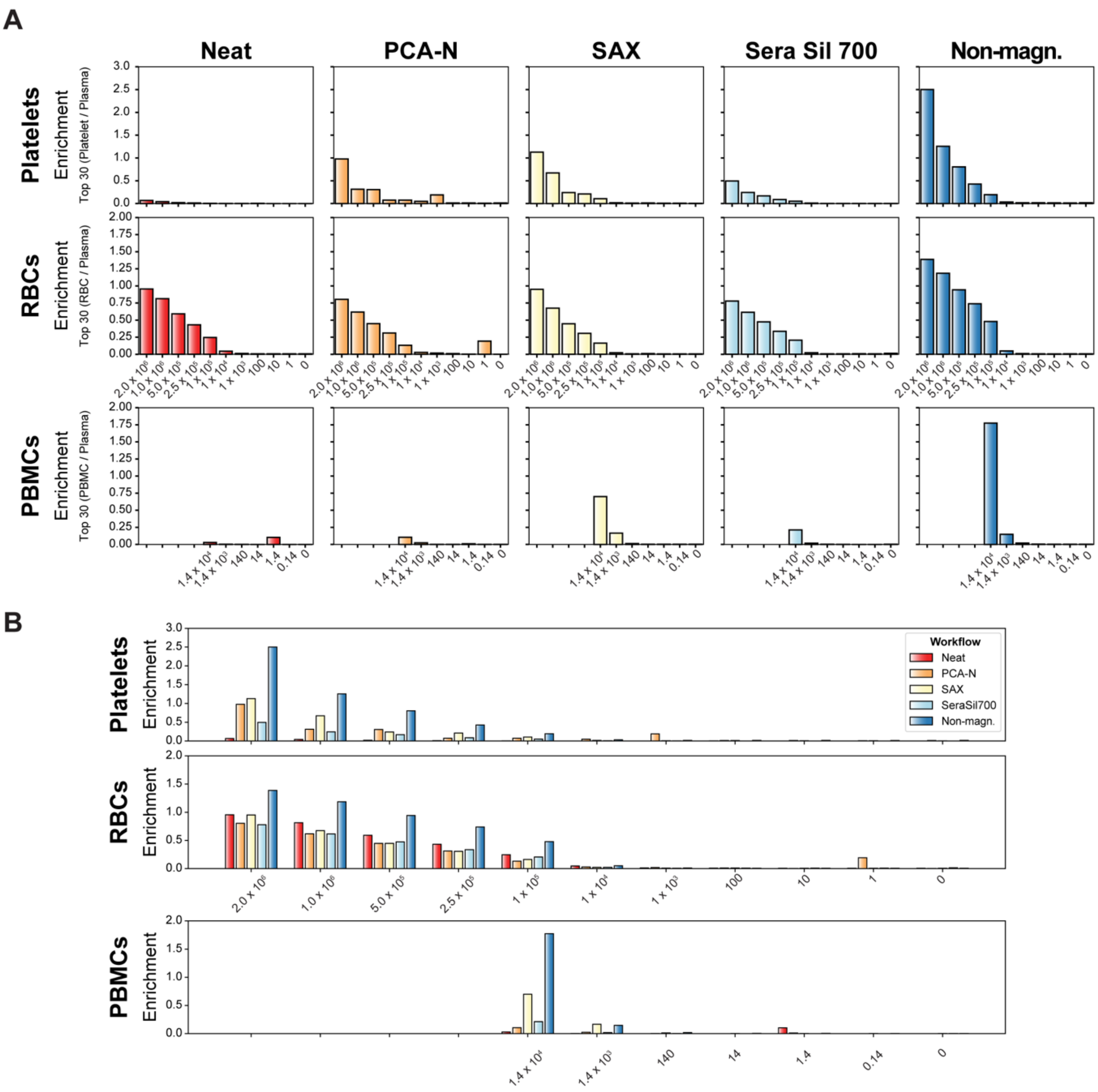

*Appendix Figure S3 - **Enrichment scores of cell-specific markers across contamination series**.*

(A) Enrichment scores for platelets (top row), erythrocytes (middle row), and PBMCs (bottom row) across all contamination levels for each of the five workflows. Enrichment score was calculated by dividing the summed intensity of the top 30 cell-specific markers by the summed intensity of the top 30 plasma proteins.

(B) Direct comparison of enrichment scores across all five workflows for platelets (top), erythrocytes (middle), and PBMCs (bottom) at each contamination level.

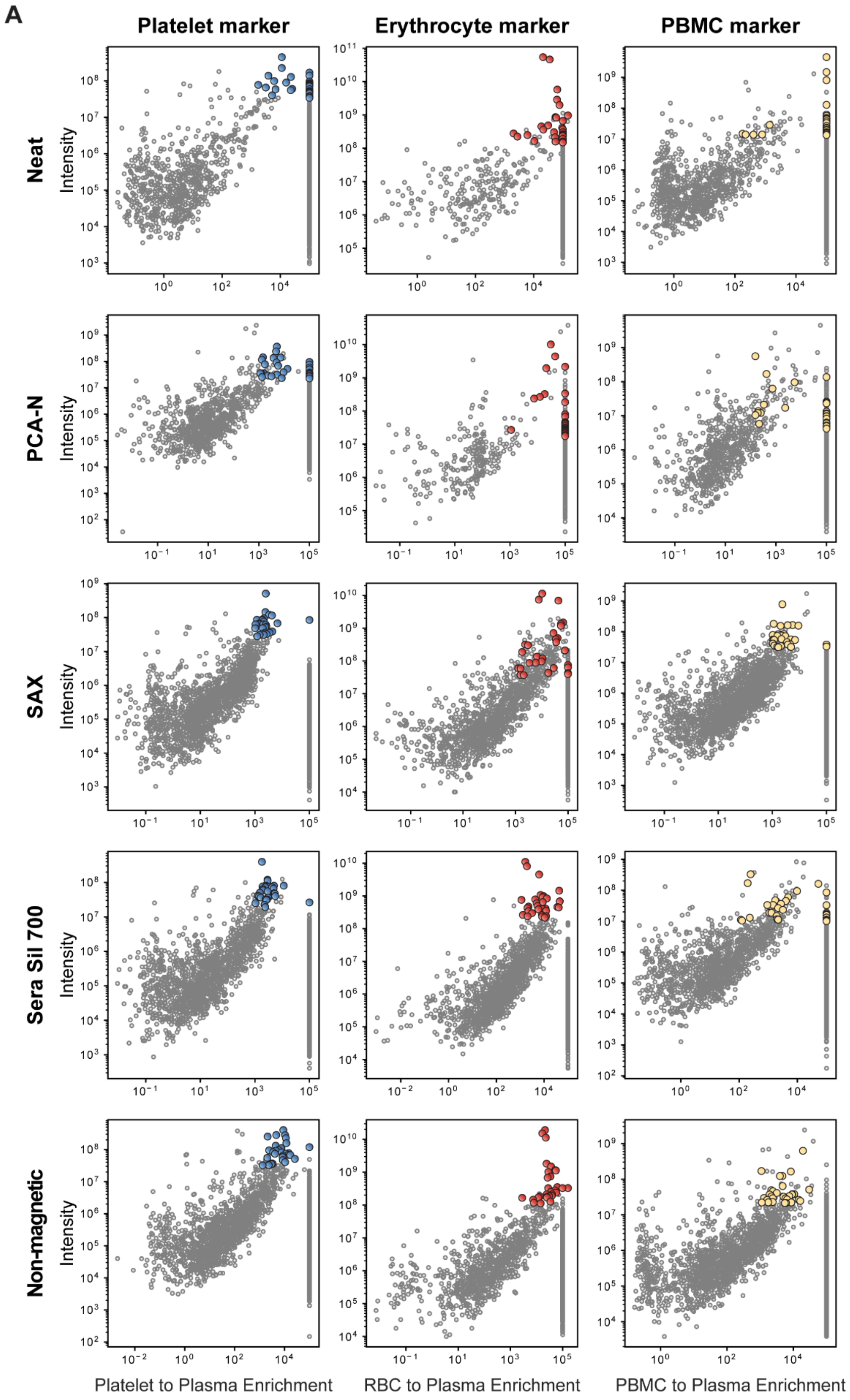
A
Platelet marker
Erythrocyte marker
PBMC marker
Neat
PCA-N
SAX
Sera Sil 700
Non-magnetic
Intensity
Platelet to Plasma Enrichment
RBC to Plasma Enrichment
PBMC to Plasma Enrichment

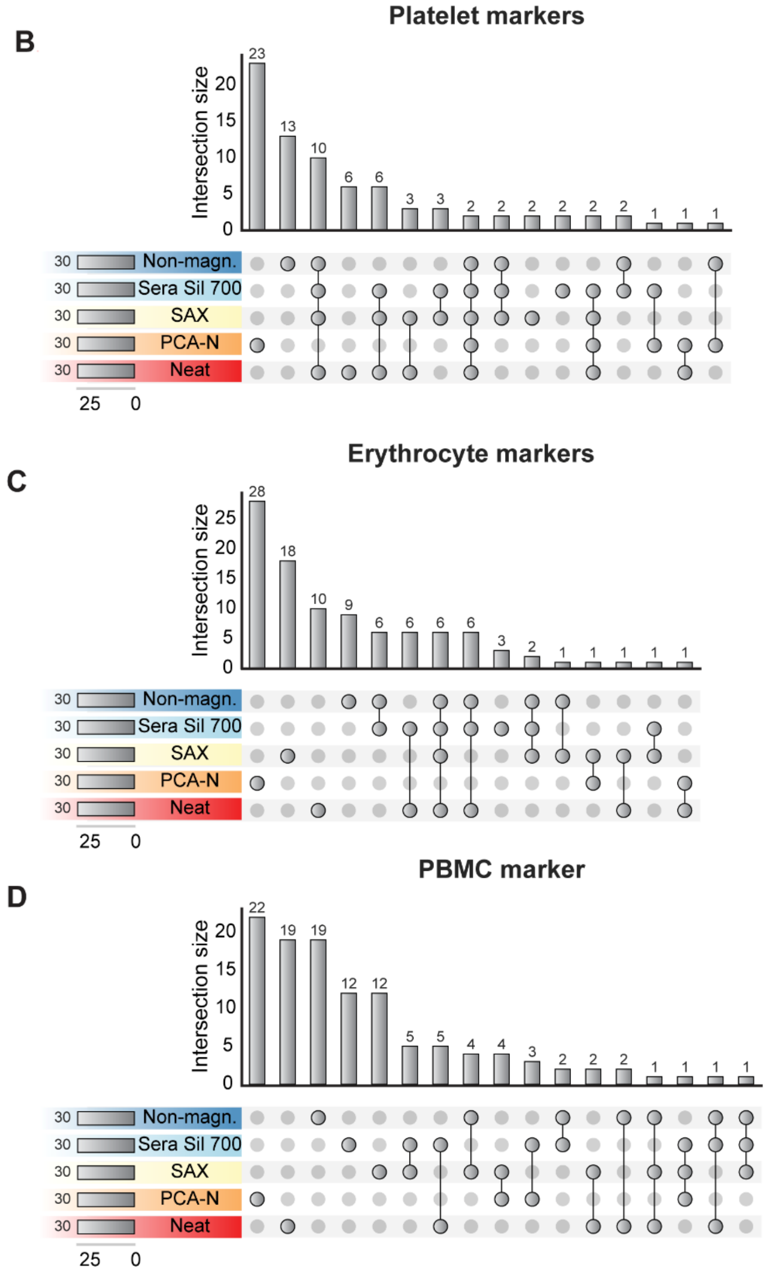
B
Platelet markers
Intersection size
0
5
10
15
20
23
13
10
6
6
3
3
2
2
2
2
2
2
1
1
1
30 Non-magn.
30 Sera Sil 700
30 SAX
30 PCA-N
30 Neat
25 0
C
Erythrocyte markers
Intersection size
0
5
10
15
20
25
28
18
10
9
6
6
6
6
3
2
1
1
1
1
1
30 Non-magn.
30 Sera Sil 700
30 SAX
30 PCA-N
30 Neat
25 0
D
PBMC marker
Intersection size
0
5
10
15
20
22
19
19
12
12
5
5
4
4
3
2
2
2
1
1
1
1
30 Non-magn.
30 Sera Sil 700
30 SAX
30 PCA-N
30 Neat
25 0

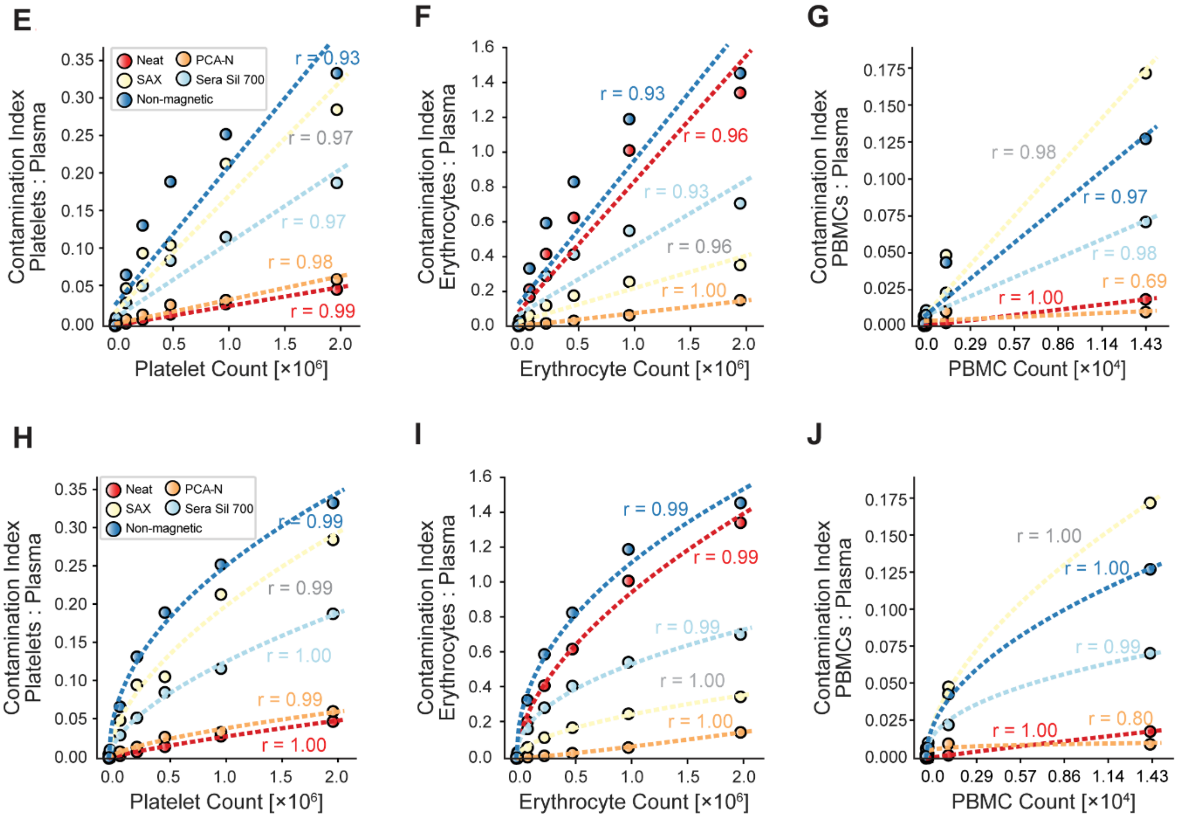

*Appendix Figure S4 - **Workflow-specific contamination marker analysis.***

(A) Contamination marker identification across workflows. Number of contamination markers identified for each cell type (platelets, erythrocytes, PBMCs) across all five workflows.

(B-D) Contamination marker overlap analysis. UpSet plots showing overlap of contamination markers between workflows for (B) platelets, (C) erythrocytes, and (D) PBMCs.

(E-G) Contamination index correlation analysis - linear model. Contamination indices plotted against cell counts with linear regression fits for (E) platelets, (F) erythrocytes, and (G) PBMCs across all workflows. Colors indicate different workflows, correlation coefficients (r) are shown for each workflow.

(H-J) Contamination index correlation analysis - power law model. Same analysis as panels E-G but fitted with power law models for (H) platelets, (I) erythrocytes, and (J) PBMCs.

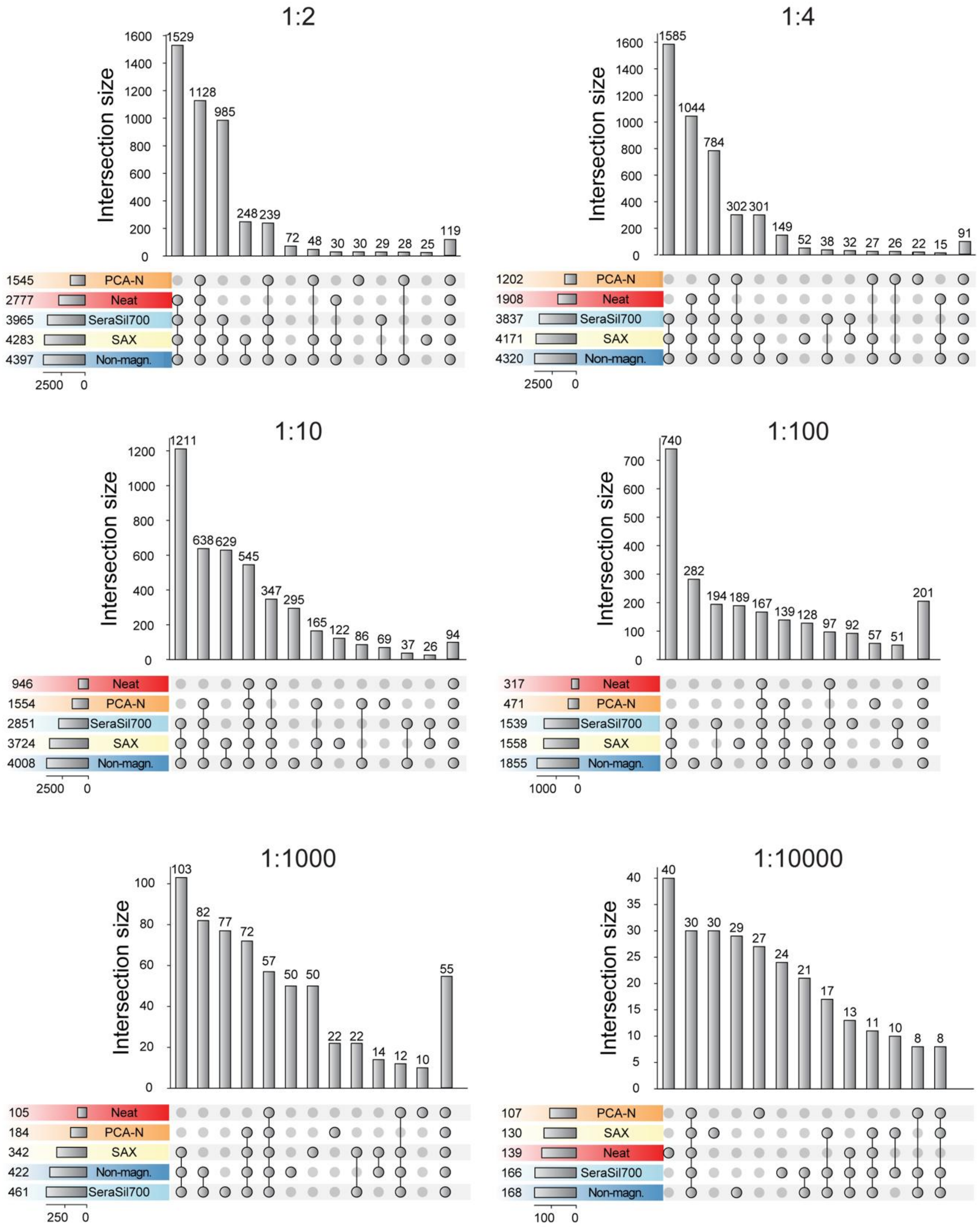

Appendix Figure S5 - ***UpSet plots showing overlap of identified yeast proteins between workflows at different dilution ratios****.*

Each panel represents a specific dilution ratio (1:2, 1:4, 1:10, 1:100, 1:1,000, and 1:10,000) with the total number of identified proteins per workflow shown in the horizontal bars on the left. The intersection size (vertical bars) indicates the number of proteins identified in each specific combination of workflows, with connected dots below showing which workflows contribute to each intersection. The total protein identifications decrease as the dilution ratio increases, with changing patterns of unique and shared identifications across workflows at different concentrations.

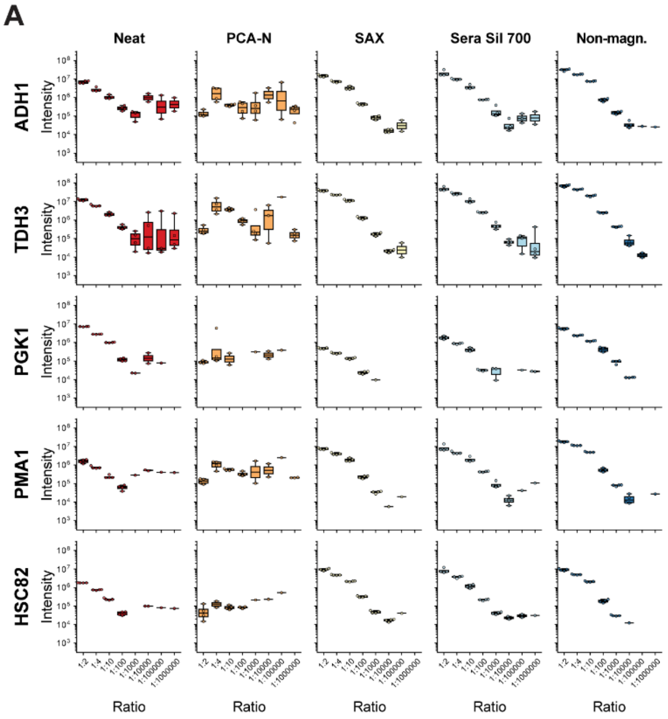
A
Neat
PCA-N
SAX
Sera Sil 700
Non-magn.
ADH1
TDH3
PGK1
PMA1
HSC82
Intensity
Ratio
1:2
1:4
1:10
1:100
1:1000
1:10000
1:100000
1:1000000

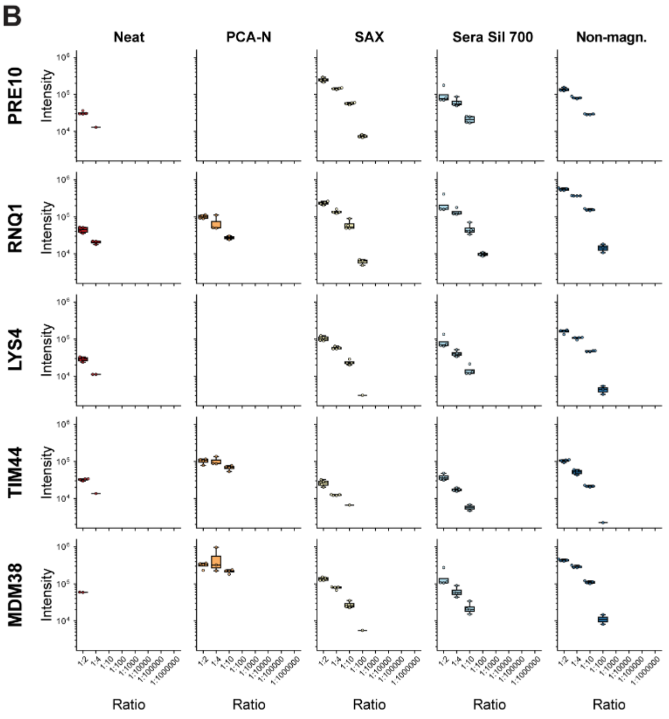
B
Neat
PCA-N
SAX
Sera Sil 700
Non-magn.
PRE10
RNQ1
LYS4
TIM44
MDM38
Intensity
Ratio
1:2
1:4
1:10
1:100
1:1000
1:10000
1:100000
1:1000000

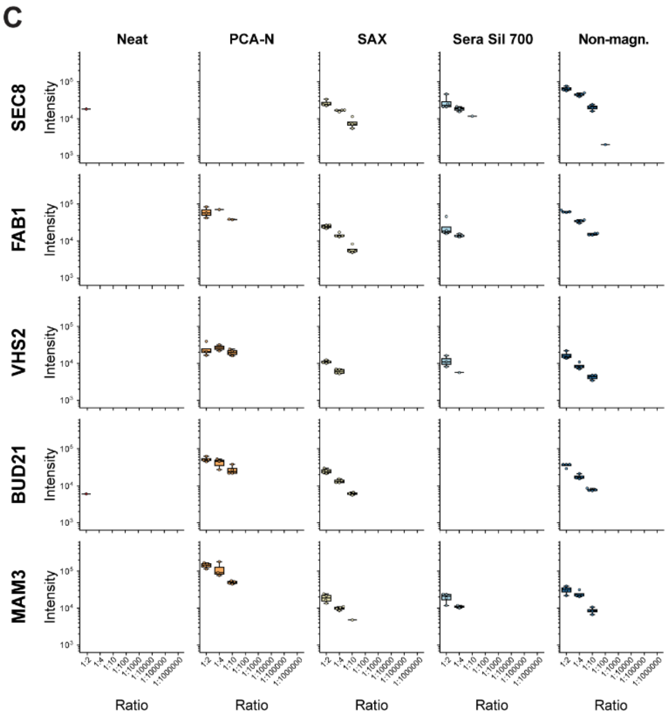

Appendix Figure S6 - ***Dilution series analysis of yeast proteins across abundance ranges.***

(A) Representative high-abundant yeast proteins showing intensity measurements across dilution series from 1:2 to 1:10^6 in all five workflows.

(B) Representative medium-abundant yeast proteins displayed across the same dilution range and workflows.

(C) Representative low-abundant yeast proteins shown across dilution series and workflows. Each panel displays protein intensity on the y-axis (log10 scale) against dilution ratio on the x-axis, with different colored lines representing each workflow's detection profile for the specific proteins.

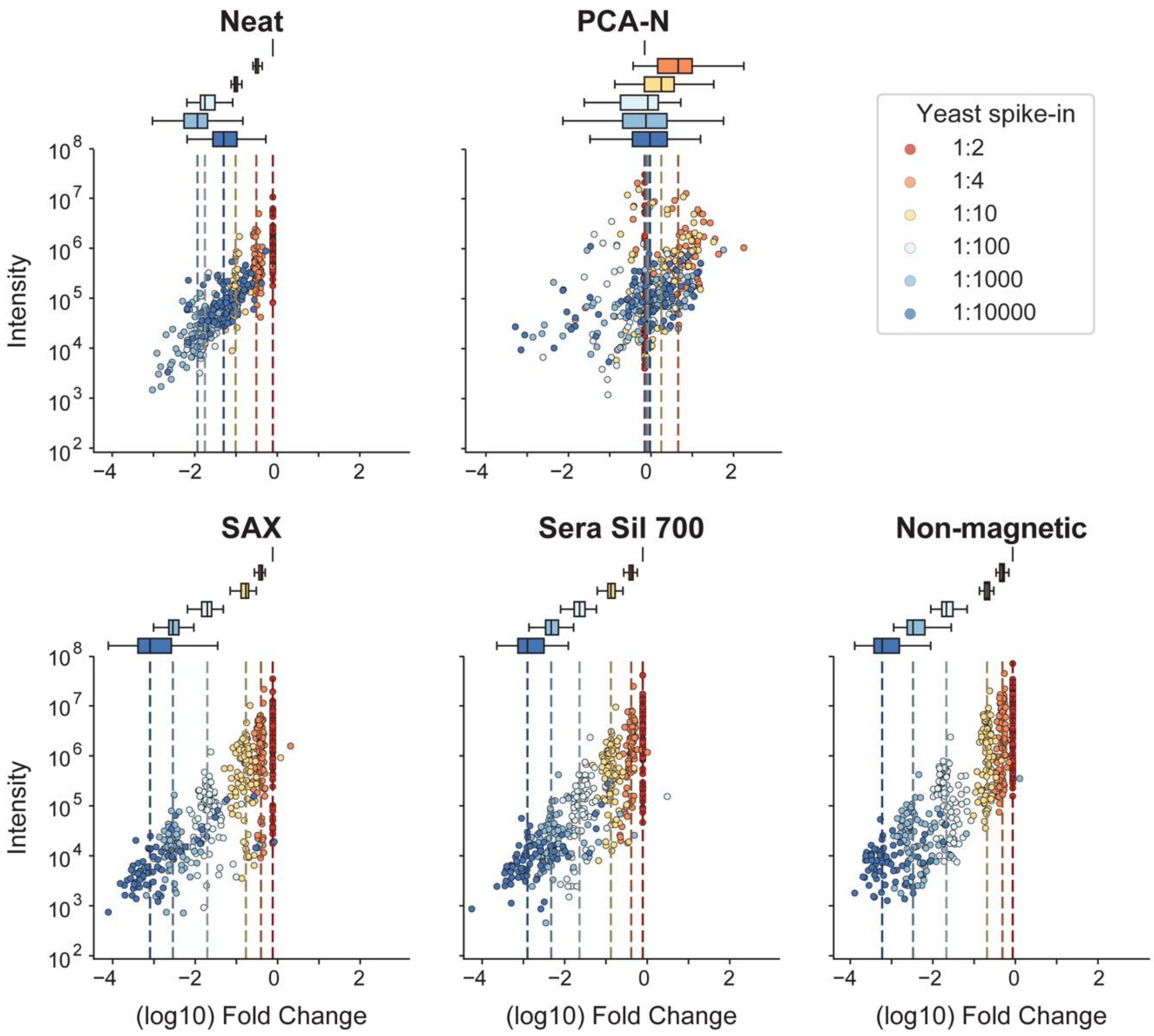

*Appendix Figure S7 - **Analysis of protein enrichment across yeast protein abundance categories**.*

Scatter plots displaying yeast protein intensity versus fold change relative to 1:2 (50%) spike-in for five plasma proteomics workflows: Neat, PCA-N, SAX, Sera Sil 700, and Non-magnetic. Data points are colored by dilution ratio. Boxplots at the top of each panel summarize fold change distributions at specific dilution points (marked by vertical dashed lines).

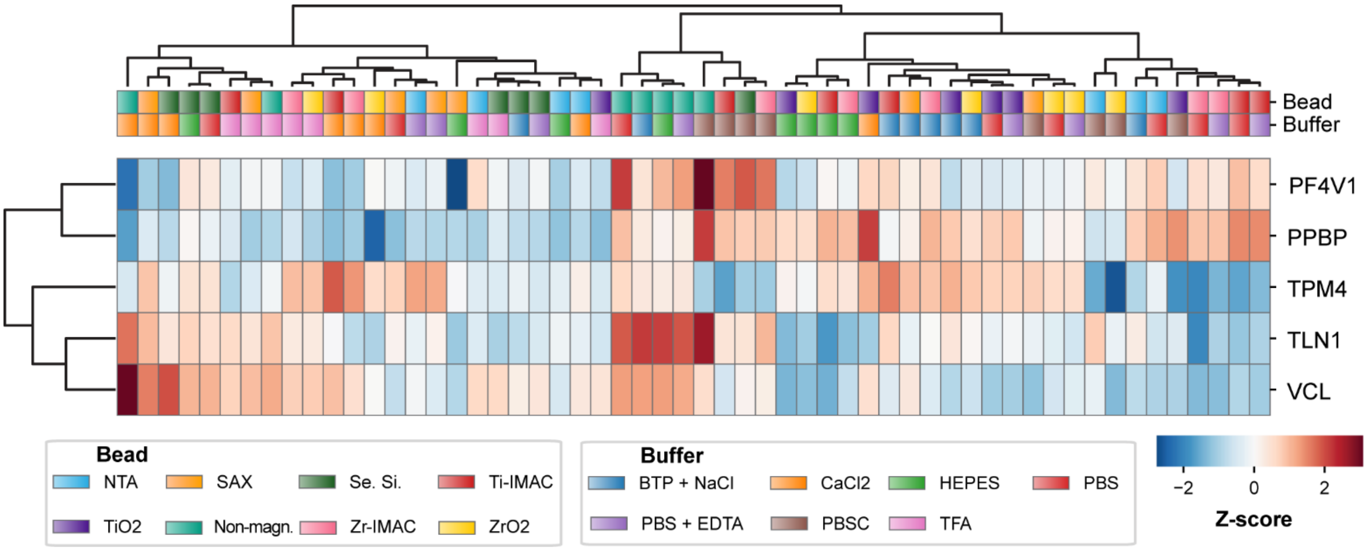

*Appendix Figure S8 - **Analysis of platelet markers across bead-buffer combinations in platelet contaminated samples**.*

Heatmap visualization of Z-scored intensities for five platelet marker proteins (PF4V1, PPBP, TPM4, TLN1, and VCL) across all tested bead-buffer combinations in platelet-contaminated plasma. Hierarchical clustering groups the markers based on similar behavior patterns across different experimental conditions. Bead types are indicated on the left and buffer conditions are shown at the bottom of the heatmap. Color intensity represents the relative abundance of each protein, with red indicating higher and blue indicating lower Z-scored intensities.

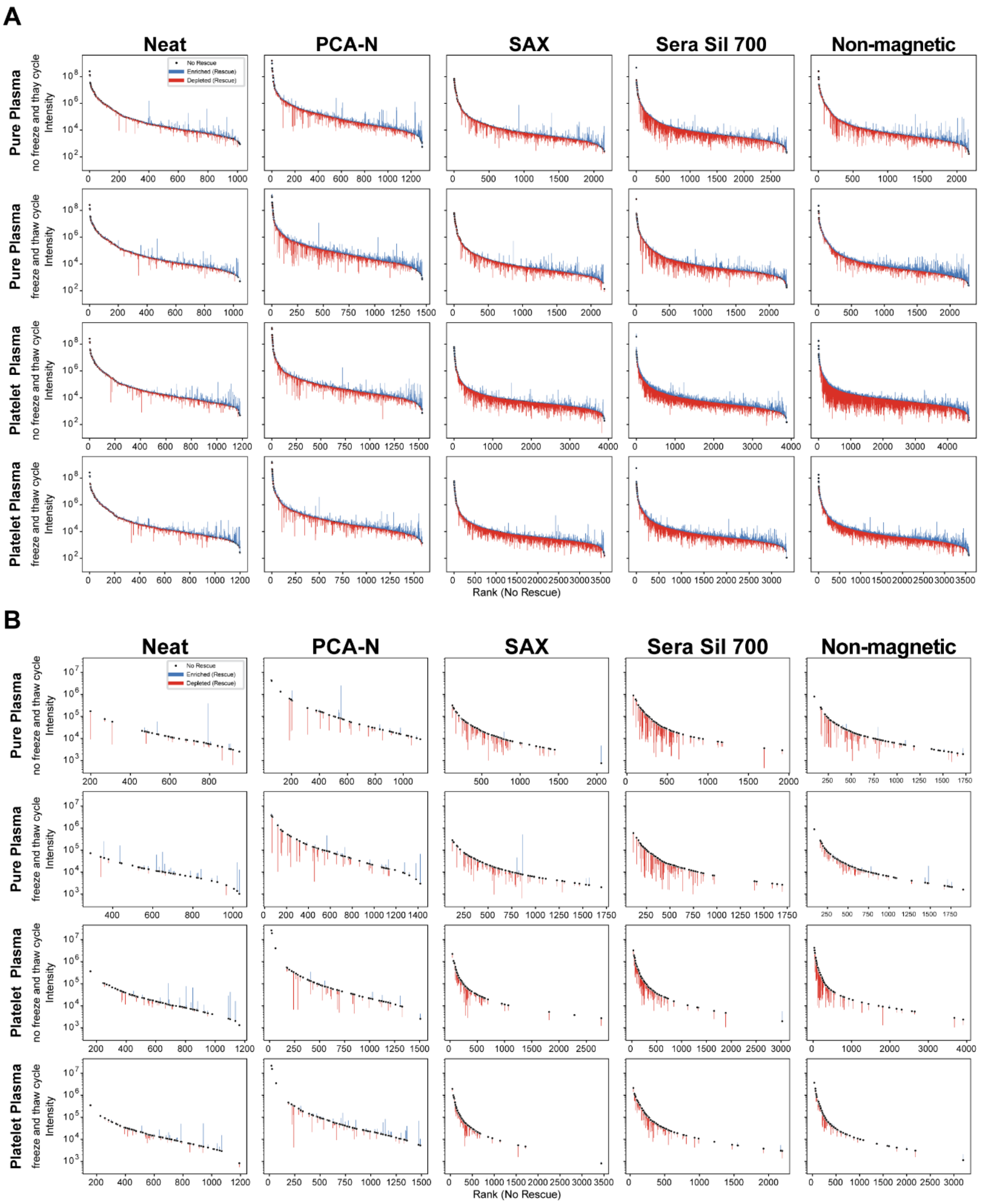

*Appendix Figure S9 - **Enrichment/depletion analysis before and after 30-minute centrifugation across workflows.***

(A) All proteins: Proteins ranked by intensity in non-centrifuged samples (black dots) with corresponding intensities after centrifugation shown as enriched (red lines)) or depleted (blue lines) across all five workflows. Each row represents different conditions, each column different workflows.

(B) Top 100 platelet markers: Same analysis focused specifically on the top 100 platelet-specific proteins.

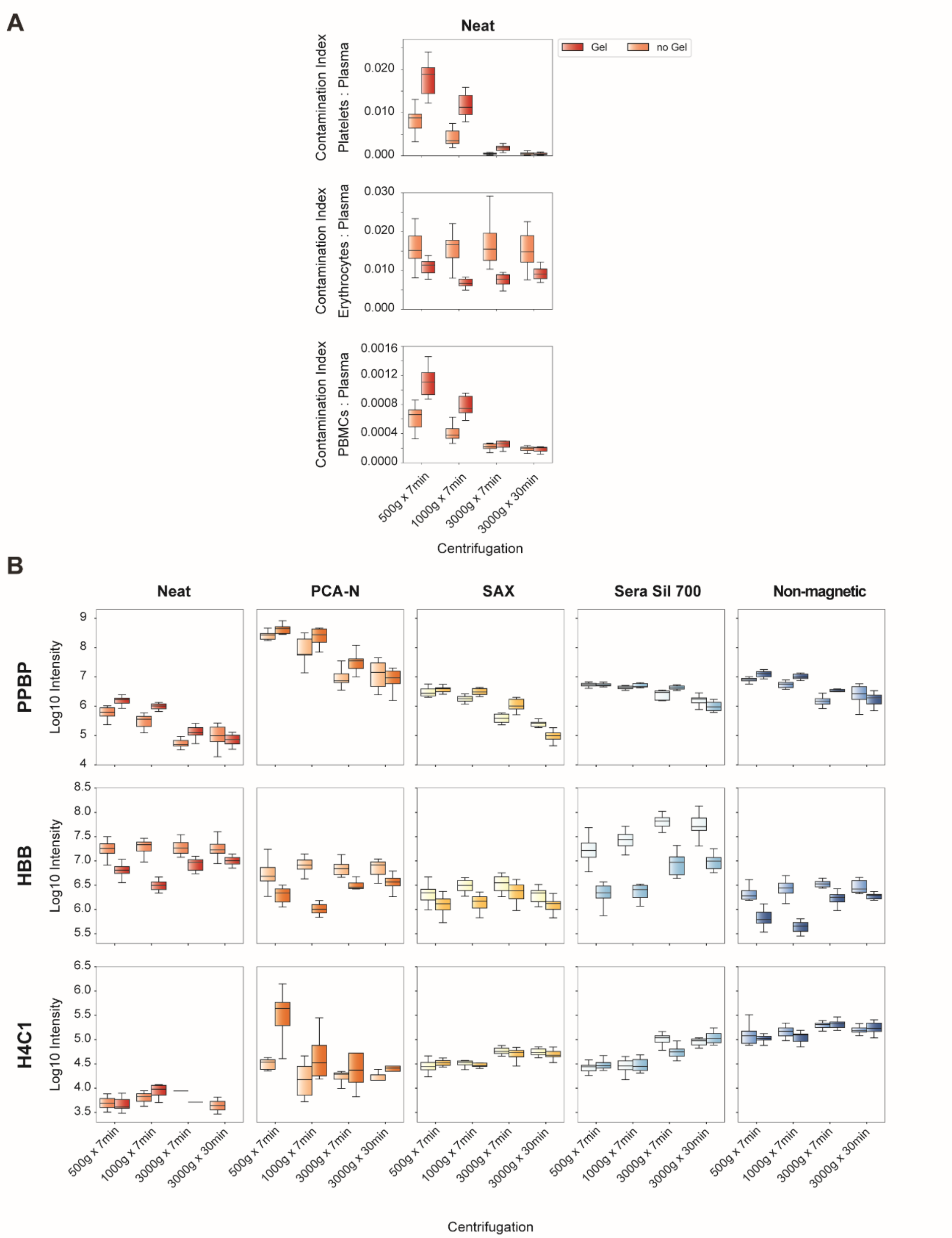

*Appendix Figure S10 - **Analysis of contamination markers across centrifugation conditions.***

(A) Contamination indices for platelets, erythrocytes, and PBMCs in the neat workflow across all centrifugation conditions, comparing gel and no-gel tubes.

(B) Abundance of specific marker proteins across centrifugation conditions and workflows. Top row: Platelet-specific marker PPBP (Platelet Basic Protein). Middle row: Erythrocyte-specific marker HBB (Hemoglobin Subunit Beta). Bottom row: PBMC-specific marker H4C1 (Histone H4). Boxplots show distribution of log10-transformed intensities across 11 individuals for each condition.
