# Supplementary material for: Pre-analytical drivers of bias in bead-enriched plasma proteomics
Source: EMBO Mol Med. 2025 Sep 12;17(11):3174–96. doi: 10.1038/s44321-025-00309-0 (PMC12603263; doi:10.1038/s44321-025-00309-0)
Supplement: Supplementary file 2 — Table EV1 [file 44321_2025_309_MOESM2_ESM.docx]

Table EV1

|  | **Neat** | **PCA-N** | **SAX** | **Sera Sil 700** | **Non-magnetic** |
| --- | --- | --- | --- | --- | --- |
| **Proteome depth** | Low | Medium | High | High | High |
| **Variability** | Low | Medium | Low | Low | Low |
| **Sample preparation duration** | Low | Medium | Medium | Medium | High |
| **Platelet susceptibility** | Low | Medium | High | High | High |
| **Erythrocyte susceptibility** | Medium | Low | High | High | High |
| **PBMC susceptibility** | Medium | Medium | High | High | High |

**Table EV1 - Summary of workflow characteristics across all tested conditions**

Comparison of the five evaluated plasma proteomics workflows across key performance and susceptibility criteria. Categories include proteome depth, technical variability, sample preparation duration, and susceptibility to cellular contamination (platelets, erythrocytes, and PBMCs). Values are semi-quantitative and based on experimental results. ‘Low,’ ‘Medium,’ and ‘High’ are relative ratings to support qualitative interpretation and workflow comparison
