# Supplementary material for: Pre-analytical drivers of bias in bead-enriched plasma proteomics
Source: EMBO Mol Med. 2025 Sep 12;17(11):3174–96. doi: 10.1038/s44321-025-00309-0 (PMC12603263; doi:10.1038/s44321-025-00309-0)
Supplement: Supplementary file 9 — Expanded View Figures [file 44321_2025_309_MOESM9_ESM.pdf]

Expanded View Figures

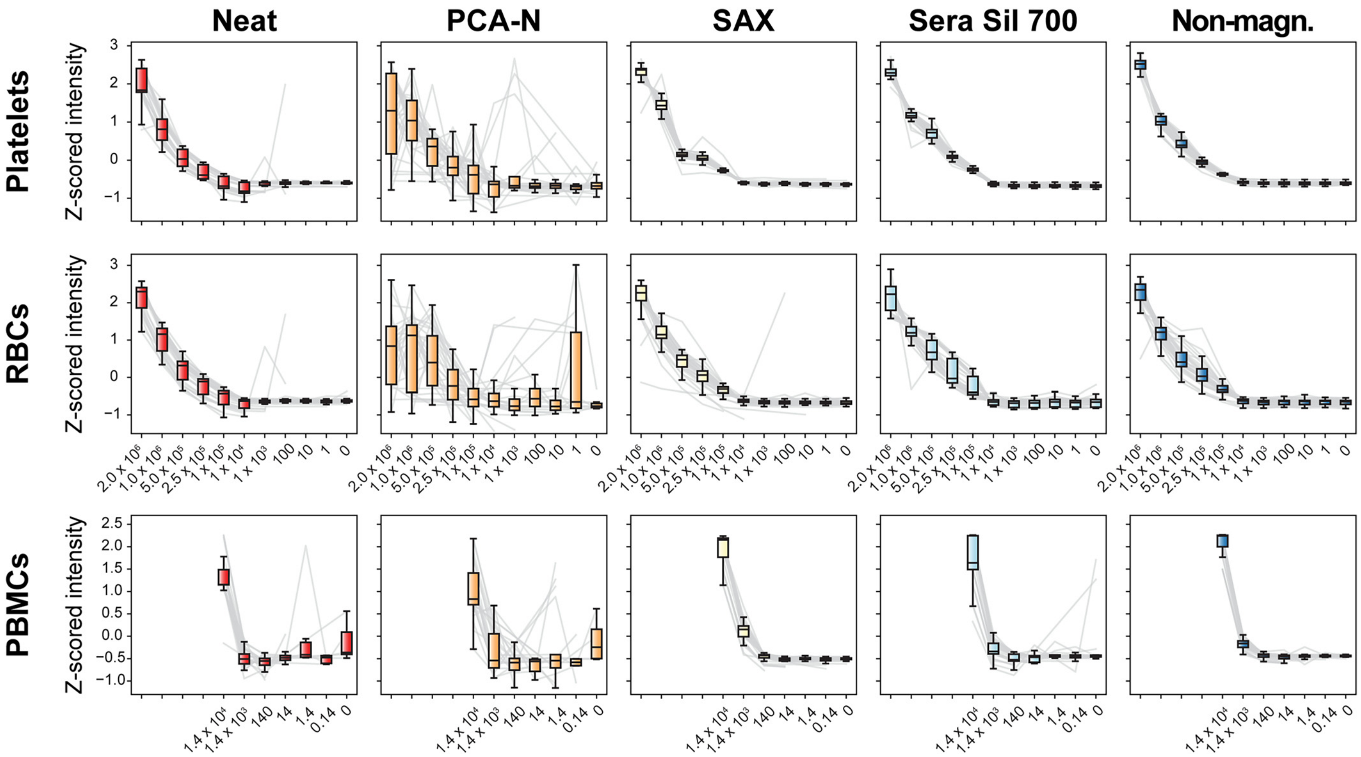

Figure EV1. Z-scored intensity profiles of cell-specific quality markers across contamination series in five plasma proteomics workflows.

Boxplots show Z-scored intensities of the top 30 quality markers for platelets (top row), erythrocytes (middle row), and PBMCs (bottom row) across all contamination levels and workflows. Gray lines represent individual marker proteins, while boxplots display the combined distribution of all 30 markers at each cell concentration. Data were Z-scored per workflow and per protein to allow direct comparison of concentration-dependent patterns.

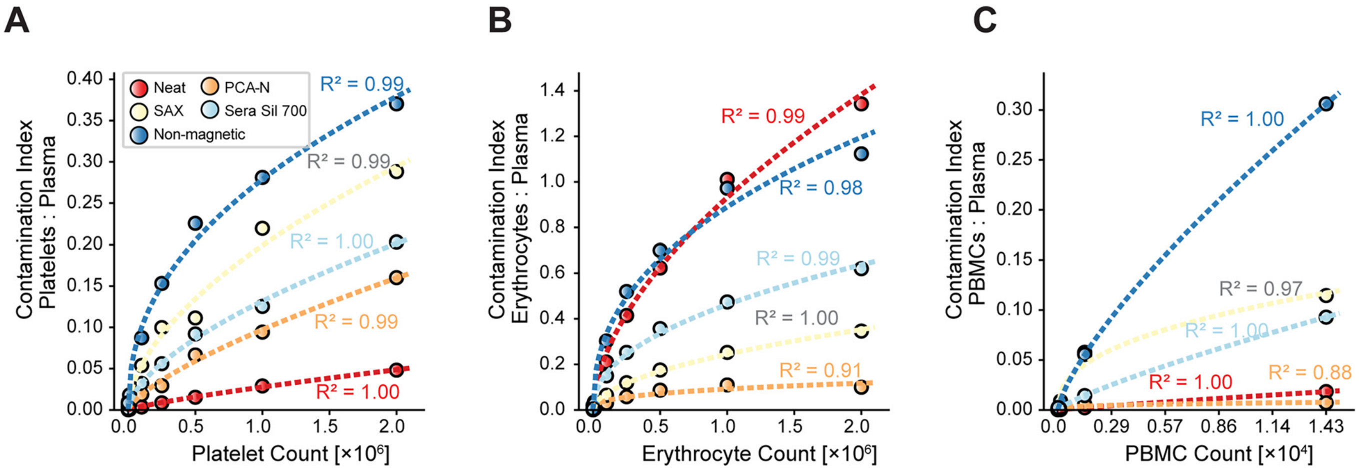

Figure EV2. Power law model fitting of contamination index data across cell types.

(**A–C**) Contamination index versus cell count with power law model fitting for (**A**) platelets, (**B**) erythrocytes, and (**C**) PBMCs across all five workflows. Points represent measured contamination indices at different cell counts, while curved lines show power law model fits. Coefficient of determination (R^2) values are displayed for each workflow and cell type.

 © The Author(s)

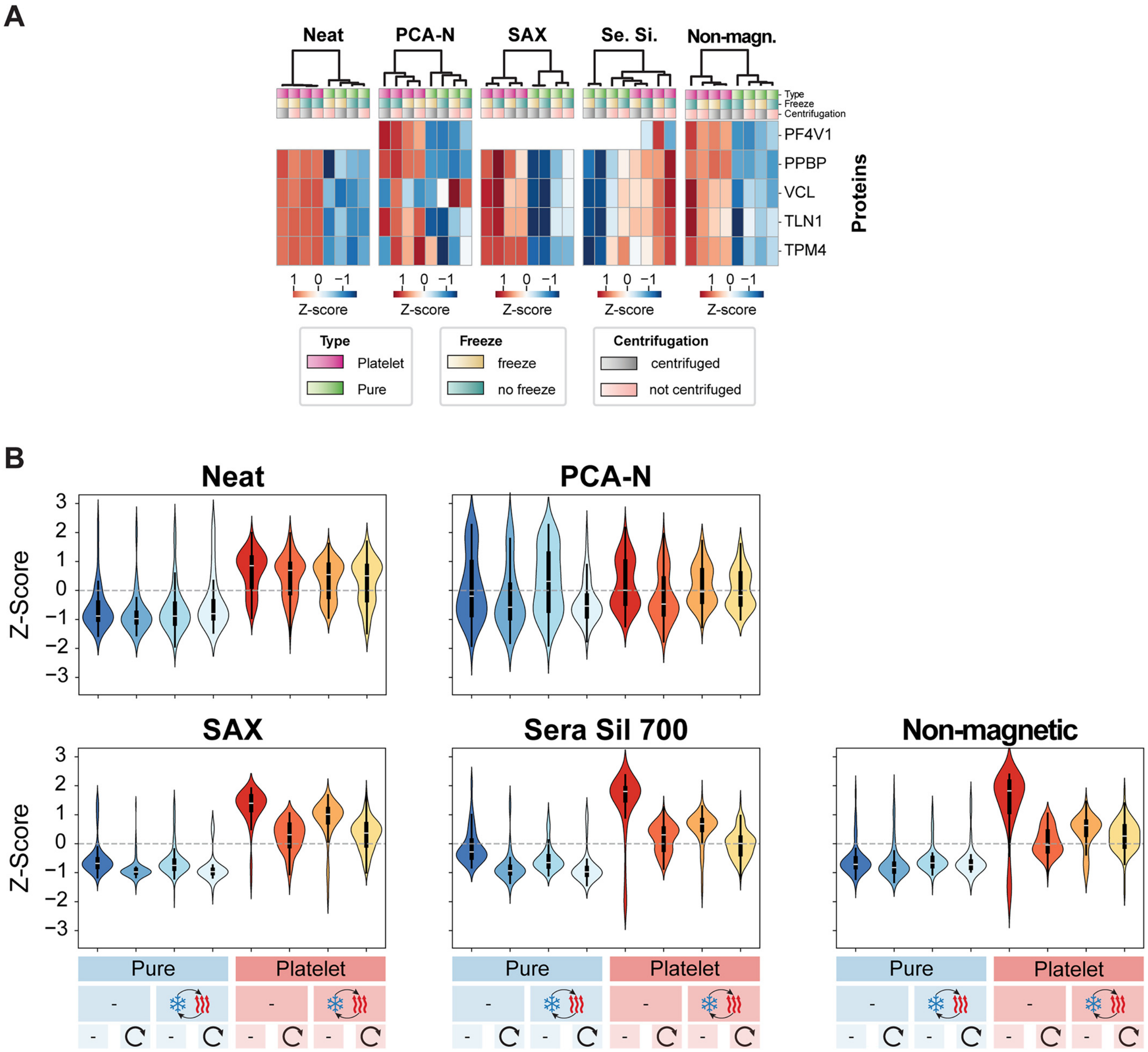

Figure EV3. Platelet marker analysis across processing conditions.

(**A**) Hierarchical clustering of five platelet marker proteins showing workflow-specific responses to processing steps. Data represent four replicates per condition. (**B**) Violin plots of Z-scored intensities for the top 100 platelet markers across different workflows and processing conditions. Blue: pure plasma; Red: platelet-contaminated. Icons indicate sample type, freeze-thaw status, and centrifugation status.

© The Author(s)

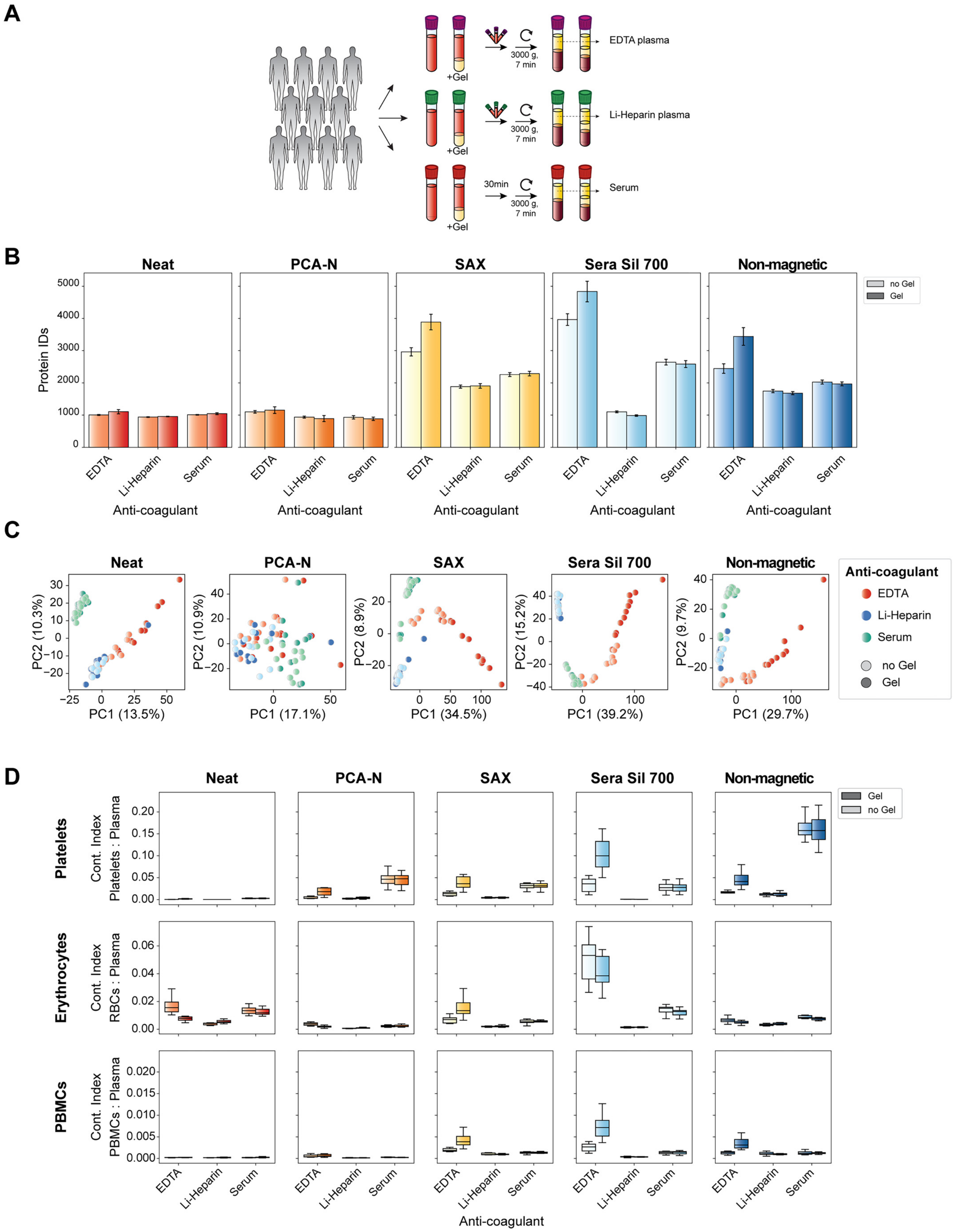
A
+Gel
3000 g, 7 min
EDTA plasma
Li-Heparin plasma
30min
Serum
B
Neat
PCA-N
SAX
Sera Sil 700
Non-magnetic
Protein IDs
no Gel
Gel
EDTA
Li-Heparin
Serum
Anti-coagulant
C
PC1 (13.5%)
PC2 (10.3%)
PC1 (17.1%)
PC2 (10.9%)
PC1 (34.5%)
PC2 (8.9%)
PC1 (39.2%)
PC2 (15.2%)
PC1 (29.7%)
PC2 (9.7%)
Anti-coagulant
EDTA
Li-Heparin
Serum
no Gel
Gel
D
Platelets
Cont. Index
Platelets : Plasma
Erythrocytes
RBCs : Plasma
PBMCs
PBMCs : Plasma

© The Author(s)

◀ **Figure EV4. Impact of anticoagulants on plasma proteome analysis.**

(**A**) Experimental design: Blood was collected from 11 healthy individuals into EDTA, Li-Heparin, and serum tubes, with and without gel separators. All samples were centrifuged at 3000×*g* for 7 min and processed through five proteomic workflows. (**B**) Number of protein identifications across five workflows for each anticoagulant with and without gel separator tubes. (**C**) Principal component analysis of proteomics data for each workflow, colored by anti-coagulant type and tube presence. (**D**) Contamination indices for platelets, erythrocytes, and PBMCs across all workflows and anti-coagulant conditions.

© The Author(s)
